# Touch and Pain Sensations in Diadynamic Current (DD) and Transcutaneous Electrical Nerve Stimulation (TENS): A Randomized Study

**DOI:** 10.1155/2019/9073073

**Published:** 2019-07-17

**Authors:** Aneta Demidaś, Mikołaj Zarzycki

**Affiliations:** ^1^Faculty of Physiotherapy, University School of Physical Education in Wroclaw, Wroclaw 51-612, Poland; ^2^School of Psychology, Bangor University, Bangor LL57 2AS, UK

## Abstract

The study investigated touch and pain sensations and the correlation between them in diadynamic current (DD) and transcutaneous electrical nerve stimulation (TENS), electrotherapies commonly applied in musculoskeletal disorders and occupational rehabilitation medicine. Forty healthy subjects were treated with either DD (n=20) or TENS (n=20). Each treatment consisted of three sessions with one-week interval. Touch sensation was determined with the JVP Domes esthesiometer, pain sensation with pressure pain threshold (PPT), and pressure pain tolerance threshold (PPTO) by an algometer. During each session the measurements were performed before the application of the procedure (T0), immediately after it (T1), and 30 minutes after the end of the procedure (T2). Both DD and TENS increased touch sensation (p<0.01) and did not significantly alter PPT and PPTO (p>0.05). No statistically significant differences in short-term effects, i.e., 3 weeks of the trial, were noted between DD and TENS in their influence on touch and pain sensations (p>0.05). There was a high significant correlation between touch and pain sensations in DD (r=0.86). TENS and DD caused similar analgesic effects. DD, which is shorter in the duration of the treatment, may comprise a realistic alternative to TENS in clinical practice of pain management.

## 1. Introduction

Diadynamic current (DD) and transcutaneous electrical nerve stimulation (TENS) are among numerous nonpharmacologic treatments applied in electromedicine, especially in musculoskeletal disorders and occupational rehabilitation medicine [1–3]. TENS is one of the most frequently used methods in physical rehabilitation with moderate and strong evidence of its ability to modulate pain. Less frequently used but incorporated in most electrical stimulator devices is DD. There are not many studies comparing the efficacy of these two electrotherapies and still they focus on the comparison of the efficacy of DD versus TENS only in terms of pain-relieving choices in physical therapy. What seems to be omitted is the case of the possible physiological mechanisms underlying the pain-relieving effects of these electrotherapies. Rehabilitation efficiency of these different methods cannot be constrained only to the examination of pain sensation. In this case the gate control theory [4, 5] constitutes a source of experimental predictions in the field of clinical rehabilitation. This theory implies that the sensitivity of the nociceptive system can be decreased or increased by gain control at the core level by the means of specific electrotherapeutic methods that increase touch sensation [6]. According to Melzack and Wall's gate control theory, both DD and TENS can influence touch and pain sensations [4, 6].

DD is considered to have a compound analgesic mechanism with the gate control system theory as its main explanation [7]. It is presumed that specific dynamics and analgesic effects include physiological processes in tissues with an influence on sensory and motor nerves. Another theory explaining analgesic mechanism of DD current states that this kind of electrical stimulation can generate an increase in the amount of endorphins, polypeptides responsible for pain-relief [3]. Both dynamogenic and inhibitory action of DD are used in treatment of various ailments. As the number of studies investigating DD is quite low, it is suggested that the evidence of its effectiveness remains scientifically weak. A single treatment session usually does not take longer than 12 minutes [3, 8, 9].

TENS is a low-frequency current. Several types of TENS are used in clinical practice [3]. Currently, conventional, high-frequency stimulation (HF TENS, high frequency TENS) and electroacupuncture, low-frequency stimulation (LF TENS, low frequency TENS) are usually applied. Both sorts of TENS are different in terms of current parameters used during the treatment [10]. HF TENS, in accordance with Melzack and Wall's gate control theory, selectively stimulates large, fast-conducting A*β* fibres that are responsible for touch sensation. Thus, HF TENS is considered to activate the analgesic mechanism at the spinal cord level, causing inhibitory effects on ascending path of pain. Double-blind clinical controlled research clearly indicates that analgesic effects of TENS are not a placebo phenomenon, but again the specific analgesic experimental predictions due to its pain-relieving mechanism are rarely examined and remain inconsistent [11–14]. The duration of the treatment is usually amounted to at least 20 minutes, longer than the case of DD.

The gate theory of pain pertains to a neurophysiological mechanism predictive in the results of touch and pain thresholds and thus may constitute a justification for the analgesic effect of DD and TENS. This theory demonstrates [4–6, 15, 16] that the afferent conduction of action potentials through the ascending tracts of a spinal cord (spinothalamic tract and dorsal column-medial lemniscus system) increases touch sensation, while simultaneously other pieces of sensory information—associated with pain sensation—can be modulated. Therefore, the following research questions were posed:

1. Do those electrotherapies, DD and TENS, change touch or pain sensations?
2. Is there a difference between them in their influence on the two types of sensation?
3. Are touch and pain sensations correlated in the application of both DD and TENS?

The aim of the present study was a comparison of the impact that two electrotherapies (DD and TENS) have on pain management and touch sensation. It was accomplished by an examination and comparison of touch and pain sensations and the correlation between them before and after the application of DD and TENS in terms of their efficacy.

## 2. Methods

*Participants*. Forty-four healthy participants aged 20 to 25 were recruited to take part in the study from students at the University of Physical Education in Wroclaw and the University of Wroclaw. The participants were recruited from August 2017 to November 2017. After having obtained written informed consent, all participants were randomly divided into two groups, the one receiving DD (22 participants) and the other TENS (22 participants). Randomization was prepared electronically with QuickCalcs Software (GraphPad Software, La Jolla, CA, USA). The DD group included 22 people, comprising 2 men and 20 women; 20 people (19 women and 1 man) lasted until the end of the study. The TENS group included 22 people, comprising 2 men and 20 women, among whom 20 participants (18 women and 2 men) completed the whole 3-week study. The average age for the participants was 21 years. The study was granted permission by the Ethics Committee at the University School of Physical Education in Wroclaw (No 07/04/2009).

*Experimental Design*. After having been randomly allocated to 1 of 2 groups, treated with either DD or TENS, participants took part in 3 treatment sessions. In DD group the duration of the treatment lasted 10 minutes and was a sequence of different types of diadynamic currents whose abbreviations relate to the original French terms: diphase fixe (DF, full-wave) 2 minutes, monophase fixe (MF, half-wave) 3 minutes, longues periodes (LP, long periods) 3 minutes, and courtes periodes (CP, short periods) 2 minutes. The duration of the treatment, parameters, and the sequence of DD currents were established according to the methodology of Bernard's current and were identical to the procedure employed by Ratajczak et al. [3]. The therapeutic intensity depended on the patient's individual reactions; i.e., it was based on participant's subjective opinion regarding the felt sensation defined as the sensation below the pain threshold but above the touch threshold. It was raised up to strong yet comfortable sensory threshold. In TENS group the duration of the treatment amounted to 20 minutes with a current frequency equal to 100 Hz, pulse width 150 *μ*s. The generated impulse was bidirectional, symmetrical, and rectangular with the intensity depending on the patient's individual reactions, same as the case of DD.

During each session the measurements were performed before the application of the electrotherapeutic treatment (T0), immediately after the procedure was completed (T1) and 30 minutes after the end of the procedure (T2) in every week of all 3 weeks of treatment applied (W1, W2, W3).

*Interventions*. In the study a PHYSIOMED-Expert (PHYSIOMED-Expert; Schnaittach, Germany) was used, a two-channel stimulation current therapy unit. Treatment procedures were executed individually with each participant by a trained physiotherapist with at least 10 years of professional experience in physical medicine. All intervention procedures took place in the treatment room.

In case of TENS, one electrode was placed on the dorsal side of the dominant hand within the area of distal and middle phalanges, the other on the palm of the same hand within the same treatment area. With the use of DD current, the cathode was on the palm of the dominant hand within the treatment area of distal and middle phalanges and the anode on the dorsal side of this hand. The electrodes were covered with a folic sheet and then bandaged. In each case, carbon electrodes of the same size (6x6 cm) were used.

The aforementioned treatment parameters were determined in accordance with the theoretical guidelines for analgesic action mechanisms in relation to the gate control theory [4–6, 10, 16, 17]. The electrotherapeutic treatment was carried out in a sitting position; the participant had the upper limb flexed in the elbow joint with the forearm in supination and a bandaged hand, both resting on the support (table top or desk).

*Outcome Measures*. Testing procedures were executed by the outcome assessor with a specialization in physiotherapy. The outcome assessor did not provide any intervention (which took place in the treatment room); they were only responsible for the measurements in the separate room.

The JVP Domes esthesiometer (JVP Domes, Stoelting, Wood. Dale, IL) was used to determine the level of touch sensation or tactile sensitivity [18]. Each participant had to identify the orientation of the gaps, which were presented in order of decreasing gap values and randomly positioned either vertically or horizontally. The threshold was delineated as the dome with which the participant had gained at least 75% correct response rate. The measurements of touch sensation were made on the fingertip of the index finger of the dominant hand, ipsilaterally to the treatment area. Each participant sat comfortably in a chair (with backrest) during the test with the arm and hand held in a supine position and blindfold to turn off the sense of eyesight during the test. The fingertip was tested for about 1 second and the test was repeated 15 times with each dome.

The pressure algometer was applied to determine the pressure pain threshold (PPT), i.e., the point at which a sensation of pressure changes into a sensation of pain. The algometry pressure was measured in the force measurement unit, i.e., kilogram-force metric unit (kgf) [19]. In addition, the pressure pain tolerance threshold (PPTO) was quantified; it is the highest possible level of pain that the participant is prepared to tolerate [20]. The pressure algometer (Wagner Force TenTMFDX50; Wagner Instruments, Greenwich, CT, USA) with a 1-cm^2^ rubber tip was used to obtain the PPT and PPTO. The points were demarcated on the skin areas where the algometry was to be performed. During the study, each participant was tested with use of a pressure algometer on the fingertip of the index finger of the dominant hand.

*Statistical Analysis*. The analysis of variances with repeated measures (ANOVA) was used to compare data recorded in 3 dependent measurements during each session (T0, T1, and T2) and between all 3 weeks of sessions (W1, W2, and W3). The statistical analysis considered a time factor at 3 levels (T0, T1, and T2), a week factor at 3 levels (W1, W2, and W3), and a group factor between subjects at 2 levels (TENS and DD). Posthoc comparisons were performed with Newman-Keuls tests. Spearman correlation matrix was performed to examine the relationship between touch and pain sensitivity. The *α* level chosen for all analyses was 0.05.

## 3. Results

Results summarized in Tables 1 and 2 present that both groups showed significant increase in touch sensation globally (F=24,30; p<0,00001) and when comparing results of each time to T0 during the same session in post hoc tests (p<0,01). A statistically significant difference was observed between the basal thresholds (T0) and thresholds measured at different times (T1 and T2) indicating an increase in touch sensation. The week factor was significant at both DD and TENS (F=6,84; p<0,01). The presented results revealed no statistically significant difference between these therapies in their influence on the increase in touch sensation (F=0,43; p>0,05).

Results depicted in Table 3 show that both groups did not present significant changes in pain sensation, i.e., PPT (F=1,70; p>0,05) and PPTO (F=1,05; p>0,05). The week factor is also insignificant at both DD and TENS (p>0,05). There was no statistically significant difference revealed between these therapies in their influence on PPT and PPTO (p>0,05).

Tables 4 and 5 show the correlation matrixes between conductivity of touch and pain sensations in DD and TENS in W3. Computation of Spearman rank correlations indicated significant relationship between touch and pain sensations (p<0,05) in one of the analyzed variables in DD in W3, i.e., touch sensation in T2 and PPT in T2 (r=0,86). No significant correlations were observed in TENS in W3.

## 4. Discussion

Many studies have presented an increase in touch sensation under the influence of TENS [21–26], especially when HF TENS has been applied. In the study of Walsh et al. [17] a frequency of 110 Hz and a pulse duration of 50 *μ*s turned out to improve significantly touch sensation. If the parameters were reduced to 4 Hz and the pulse duration of 200 *μ*s, the effect disappeared. These studies confirm clinical experience, which suggests the application of a higher frequency electrotherapy in order to stimulate faster conducting sensory afferent fibres (A*β*), thus acting in practice in accordance with the gate control theory. However, in a study by Mima et al. [27], TENS frequency equal to 90 Hz caused the opposite effect, a decrease in spatial sensory discrimination. A similar effect is noticeable in studies using mechanical stimulation [28]. This discrepancy in the research is mainly explained by the diversity of methods and parameters, and in particular the frequency, which at the cellular level influences the occurrence of a subsequent excitatory or inhibitory potential depending on its initial value [29]. In the present study both therapies can be considered effective in their influence on the increase in touch sensation. The effects are observed not only at a time factor (T0, T1, and T2) during measurements in every session, but touch sensation was significantly altered when comparing results of each week to the first week. The results conform to clinical predictions of the gate control theory [4–6, 15, 16]. It can be noted that DD stimulates the mechanoreceptors with the same efficiency as the more widely used TENS.

In opposition to the predictions of the gate control theory, results in pain sensation in this study cannot confirm the hypotheses of pain reduction. There are many factors that could underlie those unexpected results. The authors do not believe that Melzack and Wall's theory could be erroneous about the experimental predictions; especially as in the research literature an increase in pain thresholds (that signifies analgesic effect) can be found when TENS is applied [30–33]. It is agreed that the main ideas of the gate control model about pain mechanisms still remain actual but the model is not correct in detail [34]. Among many kinds of pain this study focused on mechanical pain. It is thought that the slow C nerve fibres are responsible for this special conductivity of pain sensation [35, 36]. Pressure algometry is considered to measure mechanical pain and thus pain thresholds which reflect the pain sensation conducted by the nociceptive C fibres. Gate control theory suggests that a nociceptive conductivity is modulated by the conductivity of A-*β* fibres, which may be stimulated by HF TENS or DD. This study demonstrates a clear increase in touch sensation during and after HF TENS and DD, but the pain thresholds (PPT and PPTO) remain statistically significantly unchanged even though modulation of pressure pain thresholds could have been expected [4, 6, 15, 16, 30–33]. It may be presumed that the lack of significant changes in PPT and PPTO may derive from some disadvantages of pressure algometry. The effects of operator anticipation, operator reaction time, alignment error, and variation in indentation rate on PPT measurements comprise main flaws of algometry [37]. PPT presents large interindividual variability in healthy subjects [38]; therefore normative values have not been established until this time even though there is a call for standardization of this method [39]. Case-control studies show inconsistent results in algometry [40]. But these issues still cannot fully explain why most studies concerning TENS noted significant changes in pain thresholds. Moreover, there was no significant difference between DD and TENS in their influence on pain thresholds which shows that both electrotherapies are similar in pain management.

In general this study proves that TENS and DD are similar in terms of their efficacy in touch and pain sensations. In the Can et al. [8] study comparing TENS with DD both currents was proved to be effective when it comes to pain management. Ratajczak and colleagues [3] also concluded analgesic effects of both therapies. Forogh et al. [9] study compared immediate and medium-term effects of TENS and DD with the use of algometry. They observed an increase in PPT in both currents immediately after the application, but the effect did not last until later measurements (up to 48 hours after the application). What seems to cast a new light on the efficacy of DD vs. TENS in this study is a high significant correlation between touch and pain sensitivity (p<0,05) in DD in W3. No significant correlations can be noticed in TENS in W3. This very correlation informs a decreased touch sensation conforming to the decrease in pain sensation. It is in line with the clinical predictions of the gate control theory [4–6], implying the need to increase electrical intensity along with the duration of the treatments (in weeks) in order to stimulate mechanoreceptors which are habituated during the previous treatments. It may suggest that when a longer duration of treatment is considered DD might contribute to a higher analgesic effect than TENS. But to examine this suggestion further research is required to determine the lack of hypoalgesic effects in case of TENS and DD in the study and a high correlation between variables in DD in W3.

### 4.1. Limitations and Suggestion

The current study has only examined short-term effects of DD and TENS with specific parameters in a relatively small sample. The research was conducted with healthy participants what may also imply a difference in pain mechanisms between healthy humans and patients due to the pain model limitations. This study should be expanded further in a larger sample of participants and include patient group while taking into account the variations in the electrotherapeutic parameters.

## 5. Conclusions

This study was effective in increasing touch sensation using DD and TENS. Pain sensation was not significantly altered by these electrotherapies. There were no significant differences between two electrotherapies in their influence on touch and pain sensations. A high significant correlation between touch and pain sensations demonstrated in DD current in the third week of examination might suggest a higher contribution of DD to analgesic effect in comparison with TENS. DD may comprise a realistic alternative to TENS in clinical practice of pain management.

## Figures and Tables

**Table 1:** JVP Domes tests at different times and weeks with within-group comparisons.

Means (SD) of JVP Domes tests at different times and weeks.											Within group comparisons
	W1			W2			W3				*Week factor*
											F(2)= 6.84
											df=2
											P<0.01
	T0	T1	T2	T0	T1	T2	T0	T1	T2		*Time factor*
											F(2)=24.30
											df=2
											p<0.00001
*DD*	2.01 (0.74)	1.58 (0.62)	1.70 (0.71)	1.88 (0.58)	1.35 (0.43)	1.40 (0.54)	1.88 (0.71)	1.31 (0.56)	0.98 (0.28)	*P1*	
n=20											
	1.76 (0.68)			1.54 (0.55)				1.38 (0.64)		*P2*	

*TENS*	2.27 (0.69)	1.59 (0.50)	1.43 (0.52)	1.48 (0.61)	1.21 (0.85)	1.08 (0.49)	1.55 (0.71)	1.03 (0.54)	0.96 (0.61)	*P1*	
n=20											
	1.76 (0.65)			1.25 (0.65)			1.18 (0.64)			*P2*	

P1: P values comparing results of each time to T0 during the same session; p < 0.01.

P2: P values comparing results of each week to W1; p < 0.02.

For description of variables refer to text.

**Table 2:** Main between groups comparison (current group factor) with its interactions in JVP Domes tests.

Current group factor with its interactions	df	F	p
Current (DD vs. TENS)	1	0,4346	0,523312
Current and week factor	2	0,6062	0,554297
Current and time factor	2	0,0681	0,934354
Current with week and time factors	4	1,4336	0,238838

**Table 3:** Main statistical effects in pain thresholds (PPT and PPTO).

Pain threshold	PPT			PPTO		
	df	F	p	df	F	p
Current (DD vs. TENS)	1	1,97	0,188025	1	3,24	0,098890
Week factor	2	0,01	0,987588	2	0,16	0,851460
Time factor	2	1,70	0,204904	*2*	1,05	0,365643

**Table 4:** Spearman rank correlations in DD current in the third week.

n=20	T0 PPT	T1 PPT	T2 PPT	T0 PPTO	T1 PPTO	T2 PPTO
T0 Touch sensitivity	0,11	0,46	0,50	-0,18	0,21	0,39
T1 Touch sensitivity	0,39	0,71	0,71	-0,46	0,50	0,04
T2 Touch sensitivity	0,36	0,71	*0,86*	0,21	0,43	0,43

For description of variables refer to text. Correlation shown in italic is significant at a level 0.05

**Table 5:** Spearman rank correlations in TENS in the third week.

n=20	T0 PPT	T1 PPT	T2 PPT	T0 PPTO	T1 PPTO	T2 PPTO
T0 Touch sensitivity	0,31	0,09	-0,14	0,31	0,20	-0,14
T1 Touch sensitivity	0,43	0,14	-0,09	0,43	0,26	-0,09
T2 Touch sensitivity	0,14	0,49	0,37	0,14	0,26	0,20

For description of variables refer to text.

## Data Availability

The experimental data used to support the findings of this study are available from the corresponding author upon request.
